# The Anticancer Activity of Monosaccharides: Perspectives and Outlooks

**DOI:** 10.3390/cancers16162775

**Published:** 2024-08-06

**Authors:** Niamh McCallum, Mohammad Najlah

**Affiliations:** Pharmaceutical Research Group, School of Allied Health, Faculty of Health, Education, Medicine and Social Care, Anglia Ruskin University, Bishops Hall Lane, Chelmsford CM1 1SQ, UK; niamh.mccallum@aru.ac.uk

**Keywords:** monosaccharides, anticancer drugs, antitumour therapy, sugars, natural products, glycolysis

## Abstract

**Simple Summary:**

Despite recent advances in treatment options, such as chemotherapy, cancer continues to be the second-leading cause of death worldwide. Significant hurdles in the success of chemotherapy regimens include severe adverse effects, as well as drug resistance. As such, there is a strong requirement for novel pharmacological interventions in the treatment of cancer. Natural products, such as monosaccharides, are a promising potential treatment option for cancer due to their low toxicity, high solubility, and high specificity for tumour cells. Several naturally occurring and synthetically modified sugars have displayed toxicity in a variety of cancer and tumour cells.

**Abstract:**

A major hallmark of cancer is the reprogramming of cellular metabolism from oxidative phosphorylation (OXPHOS) to glycolysis, a phenomenon known as the Warburg effect. To sustain high rates of glycolysis, cancer cells overexpress GLUT transporters and glycolytic enzymes, allowing for the enhanced uptake and consumption of glucose. The Warburg effect may be exploited in the treatment of cancer; certain epimers and derivatives of glucose can enter cancer cells and inhibit glycolytic enzymes, stunting metabolism and causing cell death. These include common dietary monosaccharides (ᴅ-mannose, ᴅ-galactose, ᴅ-glucosamine, ʟ-fucose), as well as some rare monosaccharides (xylitol, ᴅ-allose, ʟ-sorbose, ʟ-rhamnose). This article reviews the literature on these sugars in in vitro and in vivo models of cancer, discussing their mechanisms of cytotoxicity. In addition to this, the anticancer potential of some synthetically modified monosaccharides, such as 2-deoxy-ᴅ-glucose and its acetylated and halogenated derivatives, is reviewed. Further, this article reviews how certain monosaccharides can be used in combination with anticancer drugs to potentiate conventional chemotherapies and to help overcome chemoresistance. Finally, the limitations of administering two separate agents, a sugar and a chemotherapeutic drug, are discussed. The potential of the glycoconjugation of classical or repurposed chemotherapy drugs as a solution to these limitations is reviewed.

## 1. Introduction

Cancer is one of the leading causes of death worldwide, accounting for over 10 million deaths in 2020 alone [1]. Data from the International Agency for Research on Cancer (IARC) suggest that the global burden of cancer will increase dramatically in years to come, with the number of newly diagnosed cases estimated to rise 47% by 2040 [1]. Cancer can be defined as the deregulation of otherwise normal cellular and molecular processes, which results in the altered growth, survival, metabolism, and metastasis of cells [2]. Chemotherapy is a commonly used treatment strategy for cancer. However, a major hurdle in the success of chemotherapy regimens is drug resistance, which can result in relapse, metastasis, and death [3]. Additionally, the non-specificity of chemotherapeutic drugs can cause off-target toxicity and thus myriad adverse effects in patients, including anaemia, nausea, and anorexia [4]. As such, there is a great need for novel anticancer drugs that can offer better selectivity and cause fewer negative side effects.

**Figure 1:**
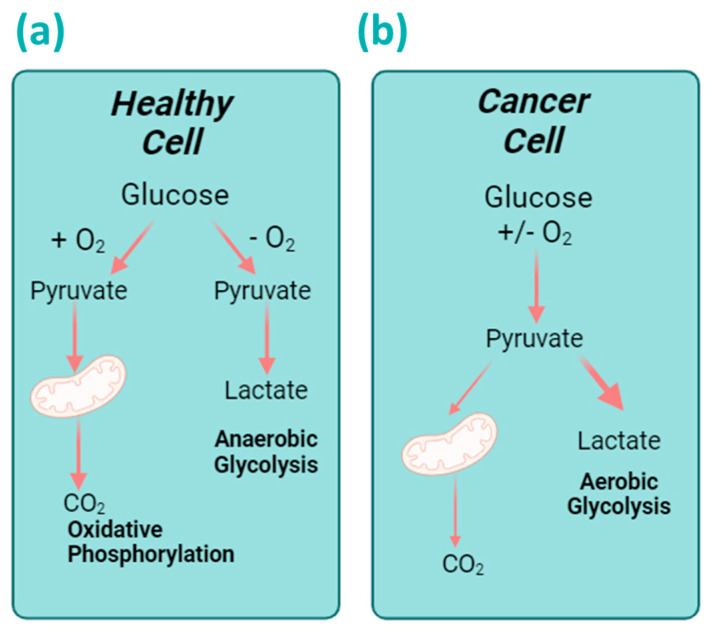
Schematic representation of the differences in metabolism between healthy cells and cancer cells. (**a**) In the presence of oxygen, healthy cells undergo oxidative phosphorylation (OXPHOS) to generate energy. Meanwhile, under hypoxia, normal cells undertake anaerobic glycolysis for metabolism, which generates lactate as the byproduct. (**b**) On the other hand, cancer cells preferentially undertake glycolysis for metabolism under both hypoxic and normoxic conditions, a phenomenon known as the Warburg effect. Created with Biorender.com.

One of the hallmarks of cancer is the reprogramming of cellular metabolism from oxidative phosphorylation (OXPHOS) to glycolysis [2]. Whilst OXPHOS is the more efficient metabolic pathway, glycolysis meets the vast and rapid energy demand of cancer cells and allows for sustained cell proliferation [5]. Increased rates of glycolysis in cancer cells are achieved via the upregulation of the glycolytic flux and overexpression of both glycolytic enzymes and the glucose transporter (GLUT) family of integral membrane proteins. This facilitates the uptake of glucose and other aldoses [6,7]. In cancer, glycolysis can occur under normoxic or hypoxic conditions, an observation known as the Warburg effect (Figure 1) [8]. Here, cancer cells consume glucose at a much higher rate than in healthy cells and secrete glucose-derived carbon as lactate [8]. Due to the high affinity of cancer cells for glucose, the use of monosaccharides to diagnose, target, and treat cancer has been explored in recent years. In fact, due to its higher rate of uptake in cancer cells, the radiolabelled glucose analogue, 2-deoxy-2-^18^fluoro-ᴅ-glucose (^18^FDG), is used in the diagnosis of tumours and in the assessment of their degree of metastasis [9].

Monosaccharides play an important role in the body, not only in energy metabolism, but also as building blocks for important biological structures, such as glycoproteins and glycolipids [10]. Due to their natural occurrence and non-toxicity, monosaccharides offer great potential in anticancer drug development. Scientists have aimed to exploit the Warburg effect by developing monosaccharides that can interfere with glucose metabolism. These compounds, known as glycolytic inhibitors, are recognised by the GLUT transporters. Glycolytic inhibitors interfere with glucose metabolism since they cannot be fully metabolised and so block glycolysis. This depletes cells of ATP energy, inducing stress and cell death, which disrupts cancer cell proliferation and migration [11]. Some sugars can inhibit glycolysis because they are epimers or derivatives of glucose, whilst glycolytic inhibitors include the synthetic glucose analogues, 2-deoxy-2-fluoro-ᴅ-glucose (2-FDG) and 2-deoxy-ᴅ-glucose (2-DG). Other monosaccharides induce cancer cell death via mechanisms such as interfering with protein synthesis and glycosylation, which can induce ER stress and the ensuing unfolded protein response, as well as promoting cell cycle arrest, anti-inflammatory effects, inducing apoptosis, necroptosis, autophagy and more, all of which will be discussed in the next sections.

Despite the huge potential of monosaccharides and their derivatives as anticancer agents, clinical translation has been limited. One reason for this is the high-water solubility of saccharides, which makes them prone to degradation by digestive glycosidases or clearance by hepatic receptors, lowering their bioavailability [12]. Another limitation is the lack of selectivity of monosaccharides in their anticancer effects. Glycolysis is the route by which the body produces energy when it is deprived of oxygen, for example, during exercise. Additionally, glycolysis is the main mode by which white blood cells, red blood cells, bone marrow cells, and nerve cells obtain energy [13]. Non-specifically inhibiting glycolysis could affect regular metabolic processes in healthy cells, resulting in damage to the body.

Despite the potential pitfalls of monosaccharides as cancer therapies, extensive research has been performed on sugars in cancer cells, revealing their potent cytostatic effects. This review will discuss advances in the use of monosaccharides for the treatment of cancer. Firstly, we will examine the literature on common and rarer monosaccharides, followed by an examination of the literature on synthetic and chemically modified monosaccharides. Finally, we will discuss the role of monosaccharides as an adjunct to classical chemotherapy and the potential of glycosylated chemotherapies.

## 2. Common Monosaccharides

**Figure 2:**
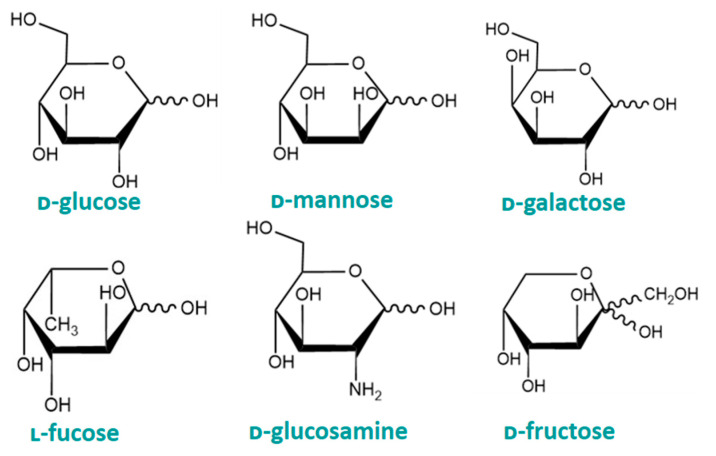
Haworth projections of the hemiacetal form of some common monosaccharides.

Asymmetry in monosaccharides gives rise to optical activity, which means that they can rotate plane polarised light either to the left (ʟ-form) or the right (ᴅ-form). ᴅ-form monosaccharides are important in the human diet since metabolic and digestive enzymes are specific for them [14]. This section will focus on the most abundant and nutritionally significant monosaccharides to the human body and how they are implicated in the progress and treatment of cancer. These include some hexoses, such as ᴅ-glucose, ᴅ-galactose, ᴅ-mannose, and ᴅ-fructose, desoxysugars such as ʟ-fucose, and the aminosugar, ᴅ-glucosamine (Figure 2).

### 2.1. ᴅ-Mannose

ᴅ-mannose is an important dietary sugar that can be obtained from fruits such as oranges, peaches, and apples [15]. As the C-2 epimer of glucose, mannose plays important roles in the human body, for example, in the glycosylation of proteins [16]. Additionally, mannose is five times more active than glucose in non-enzymatic glycation [17]. Like glucose, mannose enters mammalian cells via the GLUTs, showing no preference or specificity for uptake by any of the 15 transporters [18]. Mannose is reported to possess immune regulatory functions and can suppress inflammatory disease [19].

Crucially, mannose has displayed notable anticancer properties. For example, in A549 cells, mannose has a strong inhibitory effect on proliferation, invasion, and metastasis [20]. Recently, Gonzalez et al. studied the effect of mannose on a variety of cancer cell lines, including KP-4 pancreatic cancer cells, and found that the monosaccharide significantly inhibited cell growth in vitro [21]. Mannose treatment at 25 mM more than halved the number of live KP-4 cells compared to the control. This cytostatic effect was confirmed in vivo, where mice bearing KP-4 xenografts were orally administered mannose, and this significantly impaired and retarded tumour growth. Together, these results suggest that mannose can inhibit cancer cell proliferation both in vitro and in vivo. It was hypothesised that mannose may interfere directly with glucose metabolism, but it was found that treatment of cancer cells with mannose had no effect on intracellular glucose levels. Instead, it was discovered that mannose accumulates in cancer cells as the glycolytic intermediate, mannose-6-phosphate (M6P), which suppresses phosphoglucose isomerase (PGI), hexokinase (HK), and other enzymes involved in the metabolism of glucose, hindering glycolysis. The inhibitory effect of mannose was found to be negatively correlated to the expression of phosphomannose isomerase (PMI), an enzyme that mobilises mannose towards glycolysis by converting M6P to fructose-6-phosphate (F6P). In colorectal tumour cells (RKO), which naturally express low levels of PMI, mannose significantly inhibited cell growth [21].

**Figure 3:**
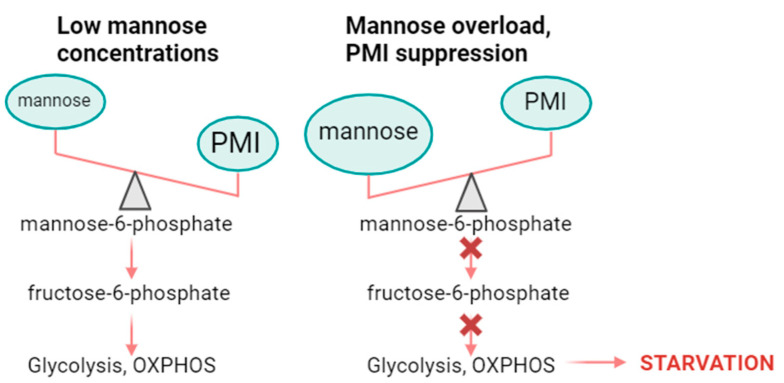
Schematic representing the effects of mannose loading on PMI processing capacity and hence metabolism. When cells are treated with mannose at doses greater than the processing capacity of PMI, less mannose is converted to fructose-6-phosphate for use in glycolysis and so metabolism is suppressed, and cells are starved of energy. Created with Biorender.com.

Saito et al. investigated the effect of mannose in leukaemia cells, which overexpress PMI [22]. Since leukaemia cells rapidly proliferate, an alternative nutrient source to glucose is often necessary for metabolism. Mannose was found to act as an energy source for metabolism in leukaemia cells, aiding glycolysis. However, when doses of mannose were used that exceeded the processing capacity of PMI, glycolysis and thus the growth of leukaemia cells were suppressed (Figure 3). These findings suggest that more evaluation is required for the use of mannose as a cancer therapy, and particular attention should be paid to the expression levels of PMI in the tumours of interest.

### 2.2. ᴅ-Glucosamine

ᴅ-glucosamine is an amino sugar that is made in the human body. Based on glucose, glucosamine is substituted at C-2 by an amine group. Glucosamine is involved in important biochemical processes, including in the synthesis of glycosylated proteins and lipids, as well as in the regulation of various signalling pathways [23,24]. An important medical use for glucosamine is in the treatment of osteoarthritis, owing to the powerful anti-inflammatory properties of this sugar [25]. Further, numerous cohort studies have found an association between habitual glucosamine supplementation and decreased cancer mortality [26,27].

The anticancer activity of glucosamine was first reported in 1953, when it was observed that in vivo administration of ᴅ-glucosamine hydrochloride to mice bearing Sarcoma 37 resulted in tumour growth retardation and the doubling of survival times of the animals [28]. In 1971, it was found that administration of glucosamine to rats bearing Walker 256 Carcinoma resulted in complete necrosis of the tumours within 40 h [29]. In 1980, Friedman and Skehan observed that treatment of C6 glioma cells with glucosamine results in cell lysis in vitro and this was potentiated by coculture with the membrane-active drug, lidocaine [30].

Despite the extensive study of glucosamine over the past 70 years, the exact mechanisms of the sugar’s anticancer activity are unclear. However, many studies have suggested that the cytotoxicity of glucosamine is related to its ability to inhibit protein synthesis and translation. For example, one study on the effect of glucosamine on DU145 and MDA-MB-231 cells found that the sugar dephosphorylated and thus inhibited the activity of the protein kinase, phosphorylated 70 ribosomal S6 protein-kinase (p70S6K), which is implicated in cell growth, proliferation, and cell cycle progression [31]. This inactivation resulted in growth inhibition in vitro, an observation that was selective to cancer cells. Similarly, Wang et al. observed an anti-proliferative effect of glucosamine (GlcN) on the human renal cancer cell lines, 786-O and Caki-1, in vitro [32]. This was attributed to glucosamine downregulating the cell cycle regulators, cyclin D1, cyclin-dependent kinase 4 (CDK4), and cyclin-dependent kinase 6 (CDK6). Meanwhile, glucosamine was found to upregulate the cell cycle inhibitors, p21 and p53. This resulted in cell cycle arrest in the phases G0/G1, as evidenced by the significantly higher volume of cells in the G1 phase compared to the S and G2 phases [32].

Glucosamine can also affect the activity of the forkhead box transcription factors (FOXOs), which modulate the expression of genes involved in apoptosis and cell cycle arrest [33,34]. Yu et al. demonstrated that glucosamine inhibited the phosphorylation of FOXO01 and FOXO03 in A549 cells, which interfered with their transcriptional activity and suppressed cellular proliferation [35]. The signal transducer and activator of transcription (STAT) transcription factors, which regulate oncogenic processes, such as proliferation and angiogenesis [36], are also affected by glucosamine. Chesnokov et al. demonstrated that glucosamine induced death in DU145 cells through the dephosphorylation of STAT3 and thus the suppression of this signalling pathway [37]. This resulted in cell cycle arrest at the G1 phase and apoptotic cell death.

A further way in which glucosamine causes an anticancer effect is through the induction of endoplasmic reticulum (ER) stress, which occurs when the ability of the ER to fold proteins becomes saturated [38]. Such stress promotes the unfolded protein response (UPR), a self-protective mechanism that shuts down protein translation and can also induce cell death via autophagy or apoptosis when stress is severe [39,40]. Hwang et al. observed reduced viability in glucosamine-treated U-87 MG cells, which was accompanied by an increase in the expression of markers of ER stress [41]. Treatment of U-87 MG cells with an autophagy inhibitor recovered cellular viability whilst treatment with an apoptosis inhibitor did not. Together, these data suggest that glucosamine induces autophagic death in U-87 MG cells via activating ER stress and triggering the UPR. Since many types of cancer are resistant to apoptosis due to mutations in the p53 tumour suppressor gene [42], targeting autophagy as an alternative cell death pathway is an important area in the development of chemotherapies [43].

Different mechanisms of action have been proposed to explain the anticancer effect of ᴅ-glucosamine; however, it remains unclear which mechanism is responsible. It is possible that a concatenation of several different effects may be attributed to the antineoplastic effects of glucosamine. Additionally, it is possible that the way in which glucosamine exerts its effects may be specific to the cancer cell line.

### 2.3. ᴅ-Galactose

ᴅ-galactose is the C-4 epimer of glucose and is one of the most abundant sugars in the human diet [44]. Galactose is widely involved in the biological function of human cells, particularly in the glycosylation of proteins and lipids, a process which plays a crucial role in cell–cell interaction, hormonal activity, immune modulation, and more [45,46,47]. In typical metabolism, galactose itself cannot undergo glycolysis so it enters the glycolysis pathway through its conversion into glucose-1-phosphate. This occurs in a series of steps known as the Leloir pathway [48,49]. However, when there is a large excess of galactose present in cells, alternative pathways can be employed in the metabolism of the sugar. One such pathway is the aldose reductase (AR) pathway, which metabolises galactose into the alcohol sugar, galactitol. Galactitol, poorly metabolised with poor liposolubility, cannot be transported across the cellular membrane. Instead, it accumulates within cells, increasing the osmotic pressure of the cells, which can result in oxidative stress and membrane rupture [50].

Exploiting the alternative metabolic pathways of galactose is one therapeutic target in anticancer drug development. Li et al. investigated the effects of galactose on the viability of some neuroblastoma cell lines (N2a, SH-SY5Y) [51]. At concentrations of 30 g/L or more, galactose inhibited the proliferation and suppressed the colony formation of neuroblastoma cells. Interestingly, galactose did not induce apoptotic cell death; instead, cell death was caused by necroptotic cell death, as evidenced by lactate dehydrogenase (LDH) leakage. Cotreatment of neuroblastoma cells with specific inhibitors of the AR pathway recovered the viability of the cancer cells, suggesting that this pathway is implicated in galactose-induced toxicity. It is possible that the leakage of LDH was a consequence of the accumulation of the AR metabolite, galactilol, leading to membrane rupture and cell death [51].

In another study, Shiratori et al. cultured A549 and HeLa cells in galactose and found that the glycolytic pathway was suppressed [52]. This metabolic suppression induced autophagy, but instead of promoting cell death, the autophagic pathway produced high levels of amino acids, shifting metabolism towards OXPHOS. This metabolic reprogramming helped in recovering the viability of the cells. It was suggested, therefore, that in some cancer cell lines, interfering with energy metabolism is not enough to cause cell death. Zheng et al. studied the effects of galactose on metabolism in breast cancer cell lines, whilst investigating whether particular oncogenes can affect the reprogramming of cellular metabolism [53]. Cells expressing AKT Serine/Threonine Kinase 1 (AKT1), an oncogene that may influence metabolism [54], were cultured in galactose-containing media, which resulted in rapid ROS-mediated cell death. This suggests that the AKT1-positive cells experienced oxidative stress and were unable to shift their metabolism from glycolysis to OXPHOS. However, after 15 days in galactose culture, the cells began to proliferate again, suggesting that the cells started to shift their metabolism and recover their viability. This study provides important evidence that certain oncogenes may expose metabolic vulnerabilities in particular cancer types. Additionally, it highlights that when developing therapies that target metabolism, the genetic profiles of tumours should be carefully considered.

### 2.4. ᴅ-Fructose

ᴅ-fructose is a simple sugar that is found in fruits and vegetables, as well as honey. Fructose is a structural isomer of glucose, and the two sugars can be converted into one another in the human body [55]. The metabolism of fructose is largely governed by fructokinase (FK), since this is the first enzyme that fructose encounters in its metabolic pathway. Fructose has a high affinity for GLUT5, and since many cancers overexpress this transporter, it is hypothesised that cancer cells may use fructose as an energy source [56,57,58].

Whilst the sugars discussed above could have potential in the fight against cancer, fructose is a far less attractive candidate. It has long been known that obesity and metabolic syndromes are associated with certain types of cancer [59] and various studies have suggested that consumption of high-fructose corn syrup (HFCS) may be responsible for the current global obesity and metabolic syndrome epidemics [60]. Furthermore, Goncalves et al. investigated the effect of high-fructose corn syrup on the growth of intestinal tumours in mice and found that consumption of just small amounts of fructose (around 3% of the total daily caloric intake of the animals) resulted in enhanced tumourigenesis [61].

In 2018, Weng et al. showed that incubation of non-small cell lung cancer, including A549 cells, with fructose resulted in enhanced proliferation of the cells [62]. The fructose-specific transporter, GLUT5, is encoded for by solute carrier family 2 member 5 (SLC2A5) [63]. Overexpression of SLC2A5, and hence GLUT5, is associated with enhanced tumourigenesis, migration, and invasion, all of which contribute to cancer metastasis [62]. In a study with MDA-MB-468 breast cancer cells, which also overexpress GLUT5 [64], fructose induced an aggressive phenotype in the cells that was associated with enhanced migratory and invasive characteristics [65]. Together, these results show that fructose is not an ideal candidate in the development of anticancer agents since it can contribute to tumour progression and metastasis.

### 2.5. ʟ-Fucose

ʟ-fucose is a dietary 6-deoxy sugar that plays a major role in mammalian biology. Unlike many of the standard hexose sugars found in the human body, fucose is mainly present in the ʟ configuration. The process of attaching a fucose unit to a molecule is known as fucosylation. Fucosylated glycans, synthesised by fucosyltransferases (FUTs), are vastly important in regulating inflammatory responses, cell signalling, transcription, protein folding, and cell–cell adhesion [66]. In cancer, fucose metabolism is altered, which can result in the aberrant expression of proteins. The potential consequences of this are evasion from immune control, invasion, metastasis, as well as reduced adhesion, and more [67,68,69]. In fact, increased fucosylation has been associated with the acquisition of metastatic properties [70,71,72]. At the same time, fucosylation is reported to reduce metastatic properties in melanoma cells [72], head and neck cancer cells [73], and hepatocellular carcinoma cells [74]. Additionally, exogenous fucose was found to rescue fucosylation in Caco-2 cells and to increase the efficiency of immunotherapy via upregulating fucosylation [75,76]. The differences in these results may be explained by the different types of tumour cells investigated in each study. Clearly, the exact extent of the protumour or antitumour effects of fucosylation warrants further exploration. As such, some researchers have investigated the effects of exogenous fucose in different models of cancer.

The effect of ʟ-fucose on solid rat mammary adenocarcinomas was first explored in a series of studies by Rosato et al. In vitro administration of fucose on mammary tumour cells resulted in significant growth inhibition in a dose-dependent manner, accompanied by morphological changes to the cells. In vivo studies showed significant reductions in the diameters of rat mammary tumours following daily IP infusion of fucose for 10 days. These early studies indicated the potential of fucose as a powerful anticancer and antitumour agent [77,78,79].

Tomsik et al. investigated the effect of in vivo-administered fucose on the growth, mitotic activity, and metastatic activity of solid Ehrlich carcinomas in immunocompetent mice [80]. IP administration of fucose significantly inhibited the growth of tumours, with a dose of 5 g/kg/day resulting in a 47% growth inhibition after 11 days. Despite this, fucose treatment displayed only a slight improvement on the survival of the tumour-bearing mice. Histological analysis of the treated tumours revealed significant decreases in mitotic indices, suggesting that fucose caused a reduction in the number of dividing tumour cells. These data suggest that fucose could be a safe and effective anticancer agent.

Recently, Yao et al. investigated whether the anticancer effect of fucose is a result of the sugar upregulating the fucosylation of proteins in cancer cells [81]. As such, the effect of ʟ-fucose on the colorectal cancer (CRC) cell line, HCT-116, a cancer accompanied with aberrant glycosylation, was studied [82]. Fucose supplementation significantly decreased the proliferation rate and the migration of HCT-116 cells, but not HCoEpic cells (healthy colonic) or HT-29 cells (CRC cells), suggesting cell-type specificity. Furthermore, fucose treatment increased the levels of fucosylation in HCT-116 cells only. Mechanistic studies revealed that fucose supplementation in HCT-116 cells upregulated the expression of genes related to serine biosynthesis, such as phosphoserine aminotransferase (PSAT1). Knocking down of this gene resulted in decreased intracellular serine concentrations, diminished fucosylation, and an impaired ability of fucose to inhibit cell migration and proliferation. Concurrently, ʟ-fucose treatment increased the intracellular concentration of serine in HCT-116 cells. This suggests that exogenous ʟ-fucose may promote fucosylation in colorectal cancer by enhancing the intracellular accumulation of serine, an important substrate in O-glycosylation [83], via the upregulation of PSAT1.

## 3. Rare Monosaccharides

**Figure 4:**
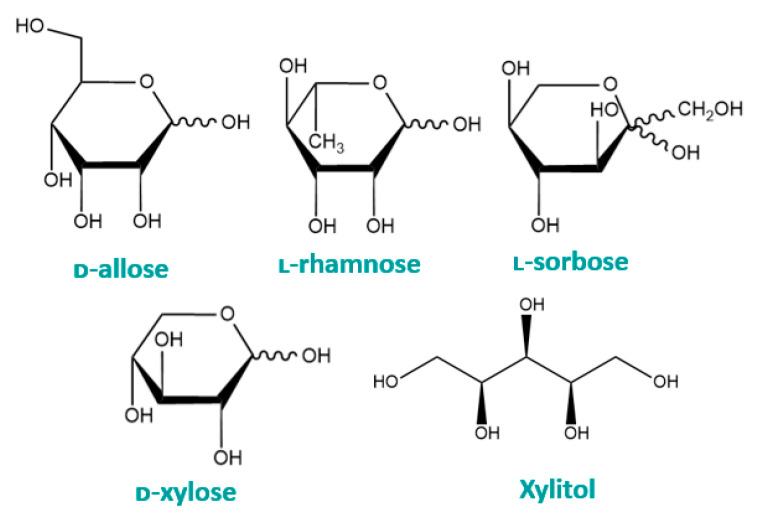
Haworth projections of the hemiacetal form of some rare monosaccharides.

Rare sugars are defined as monosaccharides and their derivatives that exist in only very small amounts in nature. The structures of some of the more widely studied rare sugars are displayed in Figure 4. According to the International Society of Rare Sugars, more than 50 types of such sugars exist [84]. Rare sugars are known to be beneficial to health, for example, in cardiovascular health and in glycaemic control [85]. Importantly, rare sugars are known to possess antitumour properties. This section of the review will assess the literature on the most widely researched rare sugars to ascertain their anticancer effects, mechanisms of action, and potential clinical applications.

### 3.1. Xylitol

Xylitol is a sugar alcohol that occurs naturally in numerous plants, including fruits and berries, mushrooms, corn husks, and the birch tree. Xylitol can also be obtained from the enzymatic or catalytic hydrogenation of ᴅ-xylose, an aldopentose [86]. The structures of these two related compounds are displayed in Figure 4. Commonly used as an artificial sweetener, xylitol is popular with diabetics and those on a low-carbohydrate diet since the sugar has a low glycaemic index and induces a low insulinaemic response [87]. In addition to being a sugar replacer, xylitol is commonly used in oral health care due to its potent antibacterial and anticariogenic properties [88]. Interestingly, xylitol is reported to suppress anaerobic glycolysis in bacteria [89]. Despite being of plant origin, xylitol can be used in mammalian glycolysis [90]. However, unlike glucose, the plant sugar generates ATP via the pentose phosphate pathway (PPP), and this is achieved through the conversion of xylitol to xylulose via xylitol dehydrogenase [91]. Due to being highly water soluble and displaying no toxicity or carcinogenicity in humans, xylitol is an attractive potential anticancer drug.

In 2014, Park et al. reported that xylitol inhibited the in vitro proliferation of a variety of human cancer cell lines in a dose-dependent manner, with the most pronounced toxic effect observed in A549 lung cancer cells [92]. The IC_50_ of xylitol in cancer cells was significantly lower than that of xylitol in healthy fibroblastic cells, suggesting some selectivity of xylitol towards cancer cells. This could be due to an overexpression of xylitol transporters in cancer cells compared to healthy cells. Mechanistic studies revealed that xylitol treatment did not induce apoptosis in A549 cells. However, when A549 cells were cotreated with the autophagy inhibitor, 3-methyladenine, the viability of the xylitol-treated cells was recovered. This suggests that the toxicity of xylitol in A549 cells is caused by the induction of autophagy [92].

More recent studies with xylitol have shown its ability to suppress glycolysis. Trachootham et al. partially substituted glucose for xylitol in the culture media of CAL-27 oral cancer cells and found that this significantly suppressed their proliferation and ATP levels [93]. Further, xylitol treatment significantly suppressed the activity of the enzyme, phosphofructokinase (PFK), a key regulator of glucose metabolism. This suggests that the sugar interfered with the glycolytic activity of the cells. This effect was not observed in healthy oral keratinocytes, suggesting a selectivity of xylitol for cancer cells. A follow-up study investigated whether the antiglycolytic effect of xylitol could be observed in vivo [94]. To achieve this, mice bearing CAL-27 xenografts were administered a glucose and xylitol-containing feed. This treatment resulted in significant retardation of tumour proliferation; however, there were no significant reductions in the volumes of the tumours. Mechanistic studies revealed that the xylitol-containing feed significantly reduced the expression of PFK, and this was accompanied by a non-significant reduction in the activity of the enzyme. This suggests that xylitol may have some effect on glycolysis in vivo.

Tomonobu et al. investigated the effects of xylitol treatment on MeWo melanoma cells in vitro and found that the sugar induced apoptosis in a dose-dependent manner [95]. Apoptosis was followed by a marked elevation in ROS and a significant decrease in the levels of reduced glutathione, an antioxidant that is involved in DNA repair. ROS were induced in cancer cell lines but not in healthy cells, suggesting a specificity of xylitol for cancer. Mechanistically, xylitol-mediated apoptosis was a result of the upregulation of CHAC1, an enzyme associated with ER stress and apoptosis, which is responsible for the degradation of glutathione [96]. In vivo, xylitol was administered to mice bearing MeWo xenografts [95]. Oral administration of xylitol had no effect on tumour volume, which was likely caused by acid-mediated inactivation of the sugar in the gut. This may also explain why in the study discussed above [94], oral administration of xylitol caused no significant reductions in tumour volumes. However, IV administration of xylitol resulted in attenuation of the tumour growth rate and significant reductions in tumour masses and volumes [95]. Furthermore, the tumour fronts of xylitol-treated xenografts were smooth, suggesting that the sugar can reduce the invasiveness of cancer cells. Mass spectroscopic analysis of tumours indicated no apparent changes in the major glycolytic metabolites, suggesting that xylitol did not affect glycolysis in vivo. However, when xylitol was co-administered with tauroursodeoxycholic acid (TUDCA), an inhibitor of ER stress, tumour suppression was inhibited, demonstrating that ER stress may be an anticancer mechanism of the sugar.

### 3.2. ᴅ-Allose

ᴅ-allose is the C-3 epimer of glucose and is rarely encountered in nature. This rare sugar is non-toxic, odourless, and highly water soluble [97]. Allose has been isolated from numerous natural resources, including potato leaves [98] and Indian seagrasses [99]. Chemical syntheses have also been employed in the production of allose; however, these are often complex reactions, with poor selectivity and unwanted side-products. Thus, enzymatic syntheses are more widely used to produce this rare sugar [100].

Despite the rarity of allose, a few anticancer studies have been conducted with the sugar. For example, allose has suppressed the in vitro proliferation of cervical and skin cancer cells [101], ovarian cancer cells [102], and prostate cancer cells [103]. Hirata et al. reported a dose-dependent inhibitory effect of allose on leukaemia cell proliferation [104]. This effect was most pronounced in the cell line, MOLT-4F. Treatment of leukaemia cells with allose resulted in cell cycle arrest, specifically in the G1 phase. This was accompanied by significantly upregulated expression levels of thioredoxin-interacting protein (TXNIP), a gene that has been reported to be a tumour [105] and metastasis suppressor [106]. Further, allose treatment significantly upregulated the levels of p27^kip1^, a cell cycle inhibitor [107]. At the same time, allose treatment did not cause apoptotic cell death, suggesting that allose does not affect the expression of proteins and genes associated with apoptosis. TXNIP is also known to regulate glucose metabolism in skeletal muscle and adipocytes [108]. Thus, allose treatment resulted in a significant decrease in the uptake of glucose into MOLT-4F cells, suggesting that the rare sugar can inhibit the metabolic activity of the cells.

Hoshikawa et al. also examined the effects of allose on TXNIP expression in cancer [109]. Allose treatment significantly inhibited the proliferation of two human oral squamous cancer cell lines in vitro (HSC-3 and Ca9-22). TXNIP expression levels were markedly increased in both cell lines, suggesting that TXNIP gene expression plays a role in suppressing cancer cell growth. This overexpression was also measured in tumour tissue following in vivo studies, in which mice bearing HSC-3 xenografts were intravenously administered a solution of allose. This resulted in a reduction in tumour volume of 39% compared to the control. These data suggest that allose, as well as other agents that can enhance the activity of TXNIP, are attractive candidates in the treatment of cancer. In addition, it was found that allose treatment significantly downregulated the expression of GLUT1 in some human cancer cell lines (HuH-7, MDA-MB-231, and SH-SY5Y) [110]. As evidenced by 2-DG uptake assays, a reduction in the expression of GLUT1 resulted in significant decreases in glucose uptake into cancer cells [110]. Together, these data suggest that allose can impart significant effects on protein expression in cancer cells, and that its consequential deleterious effect on glucose uptake is a mechanism by which the rare sugar exerts its anticancer effects.

Allose is also known to affect the expression of apoptotic proteins. Naha et al. reported an inhibitory effect of allose on the proliferation of human prostate cancer cells (DU145) [111]. Meanwhile, the rare sugar had no remarkable effect on the viability of healthy human prostate epithelial cells, suggesting a specificity of allose for cancer. Prostate homeostasis in man exists as an equilibrium between cell proliferation by mitosis and cell death through apoptosis. These activities are regulated by the antiapoptotic protein, B-cell leukaemia/lymphoma 2 protein (Bcl-2), and the pro-apoptotic protein, Bcl-2-associated X protein (Bax), respectively [112]. Allose treatment significantly decreased the expression of Bcl-2 in prostate cancer cells whilst concomitantly increasing the expression of Bax, in a dose-dependent manner. In line with this, allose caused significant apoptotic cell death in prostate cancer cells. Cell cycle analysis revealed that allose triggered the significant arrest of cells in the G0/G1 phase of the cell cycle. This is expected since cell cycle regulation and apoptosis are closely related. However, this result agrees only partly with the work discussed above by Hirata et al. [104], who showed that allose caused cell cycle arrest in the G1 phase; however, allose did not cause apoptotic cell death. These differences may be explained by the different types of cancer cell lines used.

### 3.3. ʟ-Sorbose

ʟ-sorbose is a rare natural sugar that is the C-3 epimer of ᴅ-fructose. Due to its structural similarity to fructose, sorbose is internalised into cells mainly by the fructose-specific GLUT5 transporters [113]. The presence of sorbose in foods and other natural sources is not well known; however, the sugar can be produced from the bacterial-mediated dehydrogenation of ᴅ-sorbitol, which is found in numerous fruits [114]. Sorbose is an important starting material in the manufacture of vitamin C, as well as in the synthesis of other rare sugars, such as ʟ-tagatose [114]. Research into the use of sorbose as a therapeutic agent has been largely limited to antimicrobial [115] and antifungal studies [116], as well as studies concerning glycaemic control [117].

However, a recent landmark paper unveiled the powerful anticancer properties of the sugar [118]. Sorbose was reported to impart a significant inhibitory effect on the viability of various human cancer cell lines (Huh-7, HepG2, A549, HeLa, MCF-7, and K562). Mechanistically, sorbose increased the levels of cleaved caspase 3 and the ratio of BAX/Bcl2, suggesting that the sugar can induce apoptosis in cancer cells. It was found that once inside cells, sorbose is converted into sorbose-1-phosphate (S1P) by ketohexokinase (KHK). S1P subsequently inactivates the glycolytic enzyme, HK, hindering glycolysis, inducing ROS production, and, consequently, triggering apoptosis. Additionally, S1P was found to downregulate the expression of KHK-A, an isoform of KHK that mediates the phosphorylation of proteins including p62, a selective autophagy receptor. Phosphorylation of p62 by KHK-A in regular cell metabolism results in the activation of the transcription factor, erythroid 2-related factor 2 (Nrf2), which induces ROS-fighting antioxidant genes [119]. Inactivation of KHK-A by sorbose treatment caused the attenuation of the Nrf2 pathway, thus reducing the antioxidant defence system in the cancer cells. These two inhibitory effects of S1P were found to work in synergy to induce apoptosis in cancer cells. Mice bearing Huh-7 xenografts were orally administered sorbose, which resulted in significant tumour growth inhibition and reductions in tumour volumes. Following sorbose treatment, no notable weight losses were observed in the animals and the plasma glucose and insulin levels were unchanged, showing that the sugar does not affect glycaemic indices in mice. These observations suggest that sorbose could be used safely as an anticancer drug.

### 3.4. ʟ-Rhamnose

ʟ-rhamnose is a natural 6-deoxy methyl pentose sugar that is found in plants and bacteria, but is not made in mammals [120]. Hence, this sugar is recognised in the human body as a xenoantigen. Interestingly, human serum contains a high concentration of anti-ʟ-rhamnose antibodies; thus, exposing malignant cells to rhamnose can induce an antitumour immune response in vivo [121]. Despite its non-mammalian origin, rhamnose can serve as a substrate in fucose metabolism and can be incorporated into glycosides in the human body [122]. As such, it can be hypothesised that rhamnose could negatively influence fucosylation and thus the behaviour of malignant cells. These two potential mechanisms of toxicity make rhamnose an attractive agent in anticancer drug development.

In 2014, Tomsik et al. evaluated the in vitro antitumour effect of rhamnose in Ehrlich ascites tumour cells and HL-60 leukaemia cells [122]. Surprisingly, rhamnose treatment at 20 mM caused no significant reductions in the proliferation of either cell type. Rhamnose-functionalised conjugates have also been explored for the targeted destruction of tumour cells. For example, Li et al. developed a targeted rhamnose-functionalised liposome (Rha-lip) and found it to be active against various types of cancer cells [123]. Incubation of HeLa and A549 cells with Rha-lip and human serum, a source of anti-rhamnose antibodies, resulted in significant in vitro toxicity. This suggests that for rhamnose-induced cytotoxicity to be achieved, malignant cells must be cultured in the presence of anti-rhamnose antibodies. Additionally, mice bearing 4T1 breast cancer tumours were treated with Rha-lip via IV injection, which significantly retarded tumour growth. This was accompanied by no significant weight losses in the mice treated with Rha-lip, suggesting that rhamnose-functionalised liposomes could be safe and effective antitumour agents [124].

In a different study, Tomsik et al. administered rhamnose to solid Ehrlich tumour-bearing mice via IP injection [80]. Rhamnose treatment at a dose of 3 g/kg/day resulted in significant tumour growth inhibition. Additionally, this dose of the sugar significantly prolongated the lives of the animals. Further, there was a significant decrease in the mitotic indices of the tumours following rhamnose treatment, as well as evidence of necrotic processes. These data suggest that rhamnose possesses antitumour properties. Following ex vivo analysis of the organs, rhamnose revealed no metastases, suggesting that the sugar might help to prevent tumour invasion. Interestingly, the mice used in this study were not immunised with anti-rhamnose antibodies. This suggests (1) the strain of mice used in this study (NMRI) may have naturally higher basal levels of anti-rhamnose antibodies than BALB/c mice and/or (2) the mechanism of toxicity exerted by rhamnose in this study was not related to immunomodulation. It was also concluded that rhamnose does not interfere with energy metabolism like other monosaccharides can [122]. Since rhamnose is recognised by enzymes involved in fucosylation, it is possible that rhamnose exerts its anticancer effect via interfering with fucose metabolism. However, much more research is required to elucidate the mechanism of action of ʟ-rhamnose.

The effects of common and rare monosaccharides in in vitro and in vivo models of cancer, along with their proposed mechanisms of action, are summarized in Table 1.

## 4. Structurally Modified Monosaccharides

### 4.1. 2-Deoxy-ᴅ-glucose

**Figure 5:**
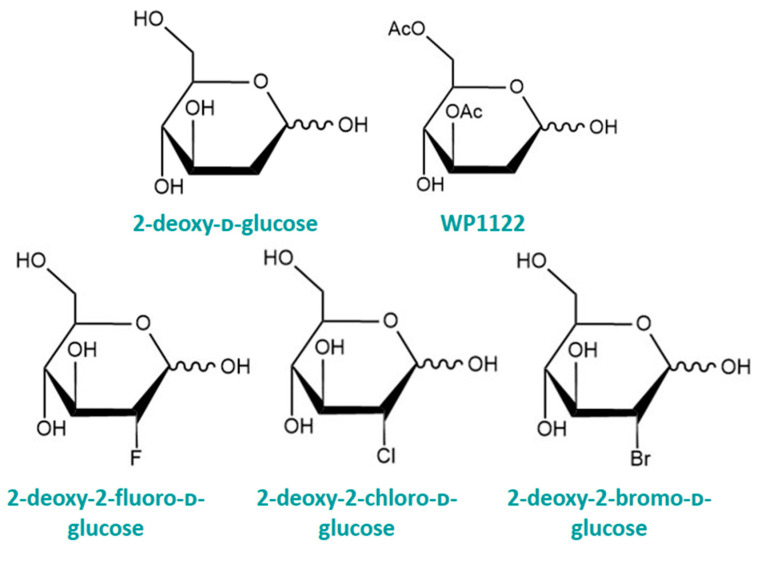
Haworth projections of the hemiacetal forms of some structurally modified monosaccharides.

2-Deoxy-ᴅ-glucose (2-DG) (Figure 5) is a synthetic, non-metabolisable glucose analogue; the hydroxyl group on carbon-2 is replaced with a hydrogen. Due to its structural similarity to glucose, 2-DG competes with glucose in the first stage of metabolism, thus inhibiting cellular ATP production [125]. 2-DG shows activity against viruses, including COVID-19, and efficacy in models of polycystic kidney disease, epilepsy, and importantly, in cancer [126]. Despite its synthetic origin, the 2-DG moiety exists in a number of natural products, including in anthracyclines and erythromycins, which may explain its vast biological applicability. Like glucose, 2-DG enters cells via the GLUT transporters and is phosphorylated to 2-deoxy-ᴅ-glucose-6-phosphate (2DG-6P) by HK [127]. However, unlike glucose, 2DG-6P cannot continue down the glycolytic pathway because it is not a substrate for PGI or glucose-6-phosphate dehydrogenase, and so accumulates in cells [127]. As such, the partial metabolism of 2-DG consumes ATP but does not contribute to the energy payoff since glycolysis cannot be completed. Studies have shown that 2-DG also affects the metabolism of glucose through competitively inhibiting PGI and so preventing the conversion of glucose-6-phosphate to fructose-6-phosphate [128], as well as non-competitively inhibiting HK [129], thus preventing the phosphorylation of glucose. This catabolic block contributes to the depletion of cellular energy.

In cancer, the inhibition of glycolysis by 2-DG is more pronounced under hypoxia. This is because, under normoxic conditions, cancer cells can flexibly shift their metabolism. However, under hypoxic conditions, cancer cells rely solely on glycolysis. In 2002, Liu et al. showed how the ATP-reducing effects of 2-DG are significantly potentiated under hypoxic conditions [130]. MIA-PaCa-2 cells were treated with 2-DG under hypoxic (<0.1% O_2_) or normoxic (21% O_2_) conditions. At a dose of 24 mM, 2-DG treatment caused ATP levels in the normoxic cells to reduce to around 60% compared to the control, whilst treatment with the same concentration of 2-DG under hypoxic conditions resulted in a reduction in ATP levels to less than 10%. These findings suggest that 2-DG is a more potent glycolytic inhibitor under hypoxic conditions. Interestingly, under hypoxia, 2-DG inhibits autophagy, which may be due to the severe depletion in ATP levels under these conditions. This may be an advantageous result since autophagy can be a pro-survival mechanism in cancer [131].

Interestingly, 2-DG can exert significant toxicity to tumour cells under normoxic conditions, suggesting that a mechanism other than glycolytic inhibition is also at play [132]. A clue to this mechanism comes from the fact that 2-DG is not only structurally similar to glucose, but it is also similar to mannose, since glucose and mannose are C-2 epimers of one another. In the 1970s, Shwartz et al. demonstrated that in certain eukaryotic cells, 2-DG inhibits N-linked glycosylation [133,134,135,136]. The authors showed how the metabolite 2-DG-GDP competes with mannose-GDP for incorporation onto N-acetyl glucosamine residues, preventing the assembly of lipid-linked oligosaccharides (LLOs). Intriguingly, the addition of exogenous mannose to 2-DG-treated tumour cells recovers their viability, which suggests that the inhibition of protein glycosylation is a major mechanism of toxicity of 2-DG [137]. Kurtoglu et al. found that inhibiting N-linked glycosylation with 2-DG results in disruption in the folding of important glycoproteins, leading to a build-up of unfolded proteins in the ER [132]. This results in the UPR, which can lead to the activation of ER stress-specific apoptotic pathways.

The UPR and autophagy are also linked, which may explain why 2-DG has been reported to induce autophagy in vitro [138,139] and in patients [138]. Xi and colleagues found that ER stress induced by 2-DG triggers calcium-mediated activation of AMP-activated protein kinase (AMPK), which leads to the stimulation of autophagy [140]. This suggests a role of AMPK as a sensor of ER stress. In addition to this, 2-DG is known to activate AMPK and induce death receptor-mediated apoptosis. Pradelli et al. observed that AMPK activation in human cancer cell lines (HeLa and U-937) is caused by glycolytic inhibition and the subsequent depletion in ATP levels [141]. AMPK activation was found to lead to the inhibition of the mammalian target of rapamycin (mTOR), a downstream target of AMPK, which can promote tumour growth [142]. Blocking this pathway resulted in the downregulation of Mcl-1, a protein of the antiapoptotic Bcl-2 family, which caused an enhancement in apoptosis. Clearly, AMPK is an important mediator of cell death and the pathway by which cancer cells take to induce death (via autophagy or apoptosis) may be cell line-dependent.

The PPP is a metabolic pathway parallel to glycolysis that regenerates nicotinamide adenine dinucleotide phosphate hydrogen (NADPH), a H_2_O_2_ scavenger, from nicotinamide adenine dinucleotide phosphate (NADP^+^) [143]. Coleman et al. treated PaCa-2 cells with 2-DG and observed depleted NADPH levels, which led to the decreased production of reduced glutathione, a major antioxidant [144]. These data suggest that 2-DG can inhibit the PPP, in addition to blocking glycolysis. Thus, 2-DG can be described as enhancing ROS levels in cancer cells in a twofold fashion: (1) by inhibiting glycolysis and reducing the levels of pyruvate, also an H_2_O_2_ scavenger, and (2) by blocking the PPP and depleting NADPH levels.

**Figure 6:**
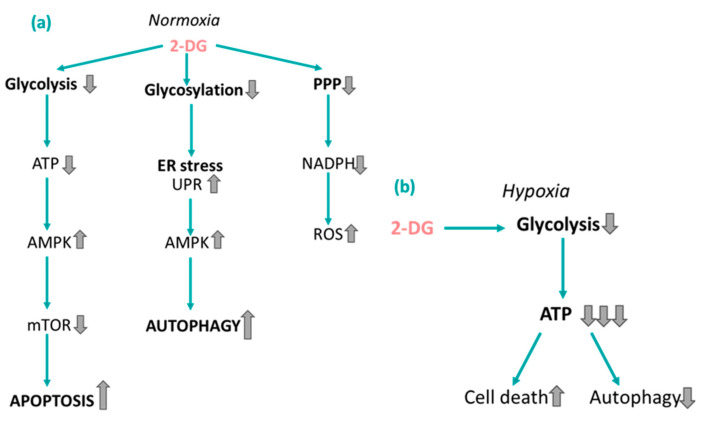
Summary of the effects of 2-DG under normoxia and hypoxia. (**a**) Under normoxic conditions, 2-DG inhibits glycolysis, reducing ATP levels, leading to apoptosis. Further, 2-DG inhibits glycosylation, which leads to ER stress and autophagy. Finally, 2-DG inhibits the pentose phosphate pathway (PPP), which results in oxidative stress. (**b**) Under hypoxic conditions, 2-DG significantly impairs glycolysis, reducing ATP levels, leading directly to cell death and the inhibition of autophagy. Upwards pointing arrows signify upregulation of a process or increased levels of a particular molecule, whilst down pointing arrows signify downregulation of a process or decreased levels of a particular molecule.

In summary, the cell-killing effect of 2-DG appears to follow a hierarchy; at low doses and under normoxia, N-linked glycosylation is inhibited, which results in ER stress and the UPR. At moderate doses of 2-DG, glycolysis is inhibited and finally, at high doses, 2-DG treatment results in the blockage of the PPP [145]. A schematic summarising the effects of 2-DG in cancer cells is presented in Figure 6. The use of 2-DG as a monotherapy in humans may be limited since higher doses of 2-DG have been linked to insulin-induced hypoglycaemic symptoms, such as flushing and sweating, during clinical trials [146]. 2-DG may be most effective when structurally modified or used in conjunction with a traditional chemotherapy, as we shall discuss in the next sections.

### 4.2. Halogenated 2-DG

The glucose analogue, ^18^FDG, was first developed in 1976 as a radiotracer to study and map glucose metabolism in the brain via positron emission tomography (PET) imaging [147]. Taking advantage of the Warburg effect and the increased demand of cancer cells for glucose, ^18^FDG and PET are now used widely as tools for localising and diagnosing metabolically active tumours. ^18^FDG differs from 2-DG in that it has an ^18^fluorine substituent on C-2, in place of the H atom. The choice to position the radiolabelled fluorine atom on C-2 is significant since substitution at this carbon does not affect the ability of the sugar to traverse the blood–brain barrier (BBB) [147].

Structurally, ^18^FDG is more similar to glucose than 2-DG. This is because the fluorine atom at C-2 is energetically and conformationally more similar to the hydroxyl group of the glucose molecule [9]. As such, ^18^FDG and its non-radiolabelled counterpart, 2-FDG, are more potent glycolytic inhibitors than 2-DG, suggesting that 2-FDG may be a better substrate for HK. Molecular modelling studies by Lampidis et al. revealed that the binding energy of the metabolite, 2-FDG-6P, for the allosteric site of HK was lower than that of 2-DG-6-P, which suggests that 2-FDG is the better non-competitive inhibitor of HK and thus glycolysis [148]. This eventuality is compounded by the fact that in experimental studies, 2-FDG more potently killed hypoxic osteosarcoma cells than 2-DG did, at the same dose. Furthermore, cells incubated with 2-FDG more potently reduced lactate levels than those incubated with 2-DG, suggesting that 2-FDG is a more powerful inhibitor of glycolysis [148]. As well as investigating the anticancer potential of the fluorinated deoxyglucose analogue, Lampidis and colleagues developed 2-chloro-2-deoxy-ᴅ-glucose (2-CDG) and 2-bromo-2-deoxy-ᴅ-glucose (2-BDG) [148]. Studies performed on the series of halogenated deoxyglucose analogues (2-XDGs) revealed that 2-FDG was the most effective at inducing cytotoxicity, followed by 2-CDG then 2-BDG. Accompanying this, 2-FDG was the best at reducing intracellular lactate levels, suggesting that 2-FDG is the most potent inhibitor of glycolysis. It appears that the cytotoxic potential of the halogenated 2-DG analogues decreases with the increasing size of the halogen substituent (Br > Cl > F). This is likely due to the decreasing ability of the 2-XDGs to effectively bind to HK as the size of the C-2 substituent increases, leading to decreased glycolytic inhibition and decreased cell killing [148]. The structures of some halogenated deoxyglucose compounds are displayed in Figure 5.

Despite the ability of 2-FDG to powerfully inhibit glycolysis under hypoxic conditions, there is little to no antiglycolytic effect and thus cell killing of the sugar when exposed to cells growing under normoxia [148]. This is because, as previously discussed, when cancer cells are grown under normoxia, they can rely on forms of metabolism other than glycolysis. One of the main mechanisms of toxicity of 2-DG is its ability to compete with mannose due to their structural similarities and inhibit protein glycosylation [132], leading to ER stress and the UPR. However, since 2-FDG has a fluorine substituent on its carbon 2 in place of a H atom, it is no longer structurally analogous to 2-deoxy-ᴅ-mannose, so has no deleterious effect on protein glycosylation. This means that it cannot exert toxicity to cells via interfering with protein glycosylation. As such, 2-FDG is a less effective anticancer agent than 2-DG.

### 4.3. WP1122—O-Acetylated 2-DG

An O-acetylated version of 2-DG, WP1122, was developed by Dr. Priebe and colleagues to enhance the efficacy of the deoxy sugar in models of glioblastoma (GBM) [149]. WP1122, also known as 3,6-di-O-acetyl-2-deoxy-ᴅ-glucose, is substituted at carbons 3 and 6 with acetoxy groups in place of hydroxyl groups (Figure 5). This chemical modification renders the sugar more non-polar, allowing it to cross the BBB via passive diffusion, as opposed to relying on uptake by the GLUT transporters [9]. Once inside cells, WP1122 undergoes deacetylation by intracellular esterases, reforming 2-DG and commencing inhibition of the glycolytic pathway. Thus, WP1122 essentially acts as a prodrug of 2-DG. Preclinical in vivo studies performed by Priebe et al. revealed that when WP1122 is delivered orally, plasma concentrations of 2-DG are twofold higher than when pure 2-DG alone is administered. Furthermore, WP1122 is well tolerated by animals and was able to significantly prolong the life of GBM-bearing mice [149]. Taken together with in vitro data that demonstrate the ability of WP1122 to effectively inhibit glycolysis under hypoxic and normoxic conditions in U-87 MG cells [149], the prodrug has excellent potential as an anticancer and antitumour agent. WP1122 is currently undergoing phase I clinical trials.

## 5. Monosaccharides as Potentiators of Chemotherapy

The combination of two or more agents in the treatment of cancer is a cornerstone of chemotherapy [150]. Conventional monotherapy non-specifically targets rapidly dividing cells, which means that healthy and cancerous cells can also be destroyed. Although combined therapies can additionally target healthy cells, their efficacy is often much greater than that of a monotherapy because the effects of the agents are synergistic or additive. Therefore, lower doses of the agents can be administered, minimising toxicity [150]. Moreover, monotherapy is more prone to drug resistance because constant treatment of cancer cells with one agent allows the cells to apply different salvage pathways [150]. As such, several conventional chemotherapeutic drugs have been tested in combination with some common sugars to inhibit cancer cell growth (Table 2).

Combination therapies with monosaccharides and classical antineoplastic drugs clearly have massive promise and could be the gold standard in the treatment of cancer. However, there are limitations to combination therapies, which include incompatible pharmacokinetics, cellular uptake, and biodistribution, as well as a potential for increased toxicity [153]. Further, given that 2-DG has been shown to cause adverse hypoglycaemic-type symptoms in clinical studies [146], it is possible that other monosaccharides that can inhibit glycolysis could do the same. Merging the two active drugs into one agent could minimise the risks associated with combination therapies.

## 6. Glycoconjugation

A glycoconjugate comprises a carbohydrate moiety, such as a monosaccharide, attached to an aglycone core via a covalent, also known as a glycosidic bond [154]. Many glycoconjugates that exist in nature possess potent cytotoxic activity and are used as anticancer agents. An example lies in anthracyclines, such as doxorubicin [155]. Glycosylation of classical chemotherapies can improve their selectivity, since cancer cells often overexpress GLUT transporters that can internalise glycoconjugates, minimising off-target toxicity [156]. Further, glycosylation can improve the pharmacokinetic parameters and expand the half-life of the drug, since one single agent is administered as opposed to two [157]. In addition, glycoconjugation can render a cytotoxic drug more amphiphilic, which is a desirable feature in drug development since this can further improve the pharmacokinetic properties and enhance cell permeation [157].

**Figure 7:**
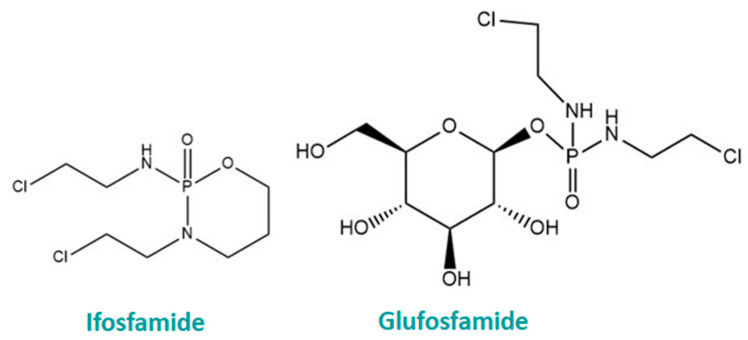
Structure of the glucose-conjugated drug, glufosfamide, compared to the structure of its parent compound, ifosfamide.

As well as those that occur in nature, numerous synthetic glycoconjugates have been developed in recent years based on classical chemotherapeutic drugs. Developed in 1995, the first GLUT1-targeting glyoconjugate was glufosfamide, a glucose-conjugated form of the cytotoxic drug, ifosfamide, as shown in Figure 7 [158]. Glufosfamide was found to be less toxic to healthy cells than its parent compound in vitro and displayed higher antitumour activity than ifosfamide in vitro and in vivo. Glufosfamide is currently undergoing phase III clinical trials, after displaying safety and efficacy in earlier trials. Other glycoconjugates based on glucose exist, including glucose–chlorambucil [159] and glucose–azomycin [160]. A potential limitation of glucose-conjugated anticancer drugs is that upon uptake into cells, the glycoconjugate may be cleaved by intracellular glucosidases, liberating glucose molecules. Since glucose is fuel for cancer cells, this may lead to cancer progression. As such, glycoconjugates based on other monosaccharides, which have antiglycolytic activity, may be better suited as anticancer agents.

In 2018, ᴅ-galactose was conjugated to doxorubicin (Gal-dox) via a small organic linker compound and was evaluated against colon cancer in vitro and in vivo [161]. The conjugate was developed to target asialoglycoprotein (ASGP) receptors, which specifically recognise galactose residues. Further, galactose prodrugs are activated by β-galactosidases, which are overexpressed in many cancers [162]. The ASGP-overexpressing colon adenocarcinoma cell line, HT-29, was treated with Gal-dox and doxorubicin and the resultant toxicities were assessed. Interestingly, both Gal-dox and doxorubicin exhibited similar toxicities to the cancer cell line. However, when the drugs were administered to ASGP receptor-negative HeLa cells, only doxorubicin, not Gal-dox, displayed significant toxicity to the cells compared to control. This suggests that targeting the ASGP receptors in colon cancer via galactose–doxorubicin conjugation not only improves the cellular uptake of the prodrug, but also enhances its cytotoxic effect. The in vivo therapeutic efficacy of Gal-dox was assessed in HT-29 tumour-bearing mice. Administration of the prodrug resulted in marked tumour growth inhibition (53.1%) compared to doxorubicin (34.9%). Despite doxorubicin being known to induce significant off-target toxicity in vivo [158], no significant toxicity was noted during this study. Together, these data suggest that Gal-dox shows enhanced selectivity and toxicity towards colon cancer compared to its parent aglycone.

Interesting examples of some glycosylated drugs are paclitaxel bound to the glycolytic inhibitor, 2-FDG (2-FDG-pax), and paclitaxel-conjugated ᴅ-glucose (Gluc-pax) [163]. 2-FDG-pax displayed increased cytotoxicity compared to paclitaxel and Gluc-pax in the lung cancer cell line, H460, and the breast cancer cell line, MCF-7. This suggests that the addition of 2-FDG and its negative effect on glucose metabolism enhances the cytotoxicity of paclitaxel towards cancer cells. Additionally, 2-FDG-pax showed less toxicity than paclitaxel or Gluc-pax to the healthy cell line, HUVEC, suggesting that conjugating 2-FDG may improve the selectivity of paclitaxel for cancer cells, improving its safety profile.

Glycoconjugates could be the next breakthrough in the treatment of cancer; however, more research must be conducted to understand the mechanisms of action of the drugs, their biodistribution in vivo, their off-target effects on healthy cells that overexpress sugar transporters, and more. Finally, more clinical studies must be performed with glycoconjugates, to assist their transition from bench to bedside.

## 7. Conclusions

In this review, we discussed the potential that common and rare monosaccharides can offer in the treatment of cancer. Monosaccharide therapies capitalise on the Warburg effect, and the accompanying overexpression of GLUT transporters, which preferentially internalise the sugars into cancer cells over healthy cells. This improves the selectivity of monosaccharides for cancer cells and reduces the incidences of off-target adverse effects. Various mechanisms have been described for the anticancer effect of monosaccharides and these include immunomodulation, anti-inflammatory effects, the inhibition of protein synthesis, which can cause cell cycle arrest, anti-migration and invasion, antiapoptotic effects, effects on autophagy, and more. Since different mechanisms are described by different researchers for the same monosaccharide, a few questions remain. Are these mechanisms occurring succinctly? Are the mechanisms reported specific to the type of cancer cells investigated? For mechanisms that have been reported based on in vitro studies, do these also occur in vivo? These are some matters that should be further investigated by research groups before more clinical studies can be performed with monosaccharides.

The main mechanism by which monosaccharides can exert an anticancer effect is through their ability to block glycolysis. Due to being structurally similar to glucose, many monosaccharides compete with glucose and begin to be metabolised in the glycolytic pathway; however, their metabolites are not substrates for glycolytic enzymes further down the pathway. Thus, these metabolites accumulate in cancer cells and prevent the generation of ATP from the metabolism of glucose. The main question posed is whether inhibiting glycolysis is enough to eradicate cancer. We have seen that blocking glycolysis can suppress cancer growth, but it still stands that cancer cells are able to use other carbon sources through OXPHOS under normoxia to produce energy. This renders cancer cells and tumours less sensitive to glycolytic inhibition than anticipated. Blocking glycolysis simply may not be enough to kill cancer. This is a major point that research groups should consider in future studies involving monosaccharides that are acting as glycolytic inhibitors.

Alternatively, combination therapies of monosaccharides and conventional chemotherapies have been shown to be more effective at eliminating cancer cells and suppressing tumours [150]. Despite this, the delivery of two distinct agents as a chemotherapy has limitations; the two drugs may have different pharmacokinetic properties and different specificities for cancer cells [153]. To address these issues, various cytostatic drugs have been conjugated to monosaccharides via direct covalent linkage, which have shown enhanced toxicity and specificity towards cancer cells compared to their parent drug.

Generally, interest in the use of glycoconjugates as cancer therapeutics has remained high in recent years. We estimate that in the near future, glycoconjugates based on potent cytotoxic drugs combined with a sugar that can inhibit glycolysis will be the silver bullet in the treatment of cancer. There has been a growing interest in the repurposing of “old” drugs to target cancer. Repurposing drugs is a potential solution to the extortionate costs, high rates of failure, and the extremely slow pace of the development of drugs and their bringing to market [164]. A glycosylated form of a repurposed drug, offering a high safety profile and high selectivity for cancer cells, could be the answer to treating this complex, heterogenous, and often multi-drug-resistant disease.

## Figures and Tables

**Table 1:** Summary of the effects of different monosaccharides and their doses on various cancer cells in vitro and in vivo, alongside their potential mechanisms of action.

Monosaccharide	Cancer Cells Tested In Vitro	Effective In Vitro Doses *	Cancer Cells Tested In Vivo	Effective In Vivo Doses	Mechanisms of Action	Refs
ᴅ-mannose	KP-4, U2OS, Saos-2, K562	10–25 mM	KP-4, K562	20–40% solution	Glycolytic suppression	[21,22]
ᴅ-glucosamine	DU145, MDA-MB-231, 786-O, Caki-1, A549, L1210, U87MG	1–5 mM	-	-	Protein synthesis inhibition, cell cycle arrest, ER stress, and autophagy	[31,32,35,37,39,41]
ᴅ-galactose	N2a, SH-SY5Y, PC-3, HepG2, A549, HeLa, PANC-1	5–333 mM	-	-	Necroptosis, glycolytic suppression	[51,52]
ʟ-fucose	Rat mammary tumour cells, HCT-116	60–300 mM	Rat mammary tumour cells, Ehrlich carcinoma	20% solution or 1–5 g/kg	Upregulation of fucosylation	[77,78,79,80,81]
Xylitol	A549, Caki-1, CAL-27, MeWo	6.5–2100 mM	CAL-27, MeWo	1–2 g/kg	Autophagy, glycolytic suppression, ER stress, apoptosis	[92,93,94,95]
ᴅ-allose	MOLT-4F, HSC-3, Ca9-22, DU145, PC-3	1–50 mM	HSC-3	2 g/kg	Upregulation of protein expression, cell cycle arrest, apoptosis	[104,107,109,111]
ʟ-sorbose	Huh-7, HepG2, A549, HeLa, MCF-7, K562	25 mM	Huh-7	20% solution	Apoptosis	[118]
ʟ-rhamnose	/	/	Ehlrich carcinoma	1–5 g/kg	Necrosis	[80]

* Dose was converted to mM for ease of comparison.

**Table 2:** Some examples of efficacious preclinical studies combining anticancer therapies with different monosaccharides.

Combination	Cell Line	Refs
Mannose + doxorubicin	KP-4	[21]
Glucosamine + doxorubicin	MCF-7/DOX	[151]
Xylitol + 5-fluorouracil	MeWo	[95]
Allose + docetaxel	HSC-3	[152]
Sorbose + sorafenib	Huh7	[118]
2-DG + paclitaxel	A549	[6]

## Abbreviations

**Abbreviations**	**Definition**
AKT1	AKT Serine/Threonine Kinase 1
AMPK	AMP-activated protein kinase
AR	Aldose reductase
ASGP	Asialoglycoprotein
ATP	Adenosine triphosphate
BBB	Blood–brain barrier
BAX	Bcl-2-associated X protein
BCl-2	B-cell leukaemia/lymphoma 2 protein
CDK4	Cyclin-dependent kinase 4
CDK6	Cyclin-dependent kinase 6
CRC	Colorectal cancer
ER	Endoplasmic reticulum
FK	Fructokinase
FOXO	Forkhead box transcription factors
F6P	Fructose-6-phosphate
FUTs	Fucosyltransferases
Gal-dox	Galactose-conjugated doxorubicin
Gal-Pt	Galactose-conjugated oxaliplatin
GBM	Glioblastoma
GlcN	Glucosamine
Gluc-PAX	Glucose-conjugated paclitaxel
GLUT	Glucose transporter
HCFS	High-fructose corn syrup
HK	Hexokinase
IARC	International Agency for Research on Cancer
KHK	Ketohexokinase
LDH	Lactate dehydrogenase
LLOs	Lipid linked oligosaccharides
M6P	Mannose-6-phosphate
MTD	Maximum tolerated dose
mTOR	Mammalian target of rapamycin
NADH	Nicotinamide adenine dinucleotide phosphate hydrogen
NAD^+^	Nicotinamide adenine dinucleotide phosphate
Nrf2	Erythroid 2-related factor 2
OXPHOS	Oxidative phosphorylation
PET	Positron emission tomography
PFK	Phosphofructokinase
PGI	Phosphogluco isomerase
PMI	Phosphomannose isomerase
PSAT1	Phosphoserine aminotransferase
Rha-lip	Rhamnose-functionalised liposomes
ROS	Reactive oxygen species
SLC2A5	Solute carrier family 2 member 5
STAT	Signal transducer and activator of transcription
S1P	Sorbose-1-phosphate
TUDCA	Tauroursodeoxycholic acid
TXNIP	Thioredoxin-interacting protein
UPR	Unfolded protein response
WP1122	3,6-di-O-acetyl-2-deoxy-ᴅ-glucose
2-BDG	2-bromo-2-deoxy-ᴅ-glucose
2-CDG	2-chloro-2-deoxy-ᴅ-glucose
2-DG	2-deoxy-ᴅ-glucose
2DG-6P	2-deoxy-ᴅ-glucose-6-phosphate
2-FDG/2-^18^FDG	2-deoxy-2^(18)^fluoro-ᴅ-glucose
2-FDG-PAX	2-FDG-conjugated paclitaxel
2-XDG	Halogenated 2-deoxy-ᴅ-glucose
**Cell Line**	**Tissue or Cell Type**
A549	Human lung carcinoma
Caco-2	Human colon carcinoma
Ca9-22	Human gingival squamous carcinoma
Caki-1	Human kidney carcinoma
CAL-27	Human squamous carcinoma
C6	Murine glioma
DU145	Human prostate carcinoma
HCT-15	Human colon carcinoma
HCT-116	Human colon carcinoma
HcoEpic	Human colonic epithelial cells
HeLa	Human cervical carcinoma
HepG2	Human liver carcinoma
HL-60	Human promyelocytic leukaemia
HSC-3	Human tongue squamous carcinoma
HT-29	Human colon carcinoma
Huh-7	Human liver carcinoma
HUVEC	Human umbilical vein endothelium
H460	Human lung carcinoma
KP-4	Human pancreatic carcinoma
K562	Human myelogenous leukaemia
L1210	Human lymphocytic leukaemia
MCF-7	Human breast carcinoma
MCF-7/DOX	Human breast carcinoma (doxorubicin-resistant)
MDA-MB-468	Human breast carcinoma
MDA-MB-231	Human breast carcinoma
MeWo	Human melanoma
MOLT-4F	Human lymphoblastic leukaemia
NCI-H23	Human lung carcinoma
N2a	Murine neuroblastoma
PANC-1	Human pancreatic epithelioid carcinoma
PC-3	Human adenocarcinoma
RKO	Human colon carcinoma
Saos-2	Human osteosarcoma
Sarcoma 37	Murine soft tissue sarcoma
SH-SY5Y	Human neuroblastoma
SK-MEL-2	Human melanoma
SK-OV-3	Human ovarian carcinoma
U2OS	Human osteosarcoma
U-87MG	Human glioblastoma
U-937	Human lymphoma
Walker 256	Murine breast carcinoma
MIA-PaCa-2	Human pancreatic carcinoma
4T1	Murine breast/mammary gland
786-O	Human kidney carcinoma
